# Transgenic expression of the human LEDGF/p75 gene relieves the species barrier against HIV-1 infection in mouse cells

**DOI:** 10.3389/fmicb.2013.00377

**Published:** 2013-12-17

**Authors:** Takuya Tada, Motohiko Kadoki, Yang Liu, Kenzo Tokunaga, Yoichiro Iwakura

**Affiliations:** ^1^Center for Experimental Medicine and System Biology, Institute of Medical Science, University of TokyoTokyo, Japan; ^2^Department of Biophysics and Biochemistry, Graduate School of Science, University of TokyoTokyo, Japan; ^3^Core Research for Evolutional Science and Technology, Japan Science and Technology AgencySaitama, Japan; ^4^Department of Pathology, National Institute of Infectious DiseasesTokyo, Japan; ^5^Research Institute for Biomedical Sciences, Tokyo University of ScienceChiba, Japan; ^6^Stem Cell Research Center, Shanghai Jiao Tong University School of Medicine Renji HospitalShanghai, China

**Keywords:** HIV-1, LEDGF/p75, IN, transgenic mouse, species barrier

## Abstract

Attempts to create mouse models for AIDS have been hampered by species barriers in HIV-1 infection. We previously showed that the nuclear accumulation of HIV-1 preintegration complex (PIC) was suppressed in mouse cells. Lens epithelium-derived growth factor (LEDGF/p75) is a host factor identified as a binding partner of integrase (IN), and has been suggested to be involved in promoting viral integration by tethering PIC to the chromatin, which are observed as nuclear accumulation of IN by LEDGF/p75. Therefore, we here hypothesized that this host factor might act as one of the species-specific barriers in mouse cells. We generated transgenic (Tg) mice that constitutively express human (h) LEDGF/p75. The GFP-fused IN was efficiently accumulated into the nucleus of hLEDGF/p75 expressing Tg mouse embryonic fibroblast (MEF) cells in contrast to the control MEF cells. Importantly, hLEDGF/p75 Tg MEF cells were significantly more susceptible to HIV-1 infection. These results suggest that LEDGF/p75 is one of the host factors that constitute species barrier against HIV-1 in mouse cells.

## INTRODUCTION

The number of patients with HIV/AIDS has been increasing throughout the world. In order to study AIDS pathogenesis and to evaluate antiviral drugs and vaccines, animal models for HIV-1 infection need to be established. A mouse model has been considered as one of such candidates because of the availability of inbred and gene-manipulated strains. As a matter of fact, mice are non-permissive for HIV-1 infection because of the species barriers against both the early, and late phases of HIV-1 infection (van Maanen and Sutton, 2003), although the precise mechanisms for the non-permissiveness remain unclear. The finding that the heterokaryons created between human and mouse cells were susceptible to HIV-1 infection (Dragic et al., 1992; Mariani et al., 2001) suggested that mouse cells lack some human-specific cofactors that can support HIV-1 replication.

A number of factors in host cells have been implicated to be involved in the early and late phases of HIV-1 infection; e.g., CD4 as the major receptor for HIV-1 entry (Maddon et al., 1985; Lores et al., 1992), chemokine receptors as coreceptors (Berson et al., 1996; Feng et al., 1996) and cyclin T1 (CycT1) for the efficient viral transcription through binding to HIV-1 Tat (Bieniasz et al., 1998; Wei et al., 1998). However, several Tg mice lines that express human versions of CD4, either CXCR4 (hCD4/hCXCR4/hCycT1 Tg) or CCR5 (hCD4/hCCR5/hCycT1 Tg), and CycT1 did not efficiently support HIV-1 replication despite HIV-1 entry and reverse transcription proceeded normally (Browning et al., 1997; Sawada et al., 1998; Mariani et al., 2000). On the other hand, Tg mice carrying the *pol *gene-deleted HIV-1 proviral genome (HIV-Tg), which we previously generated, were able to not only express all viral mRNA species, including unspliced, singly spliced, and multiply spliced mRNAs, but also produce high levels of gag p24 antigen, after treatment with bacterial lipopolysaccharides (LPS) (Iwakura et al., 1992). Taken together, these results indicate that once the viral genome is efficiently integrated into the host chromosome, viral genes are expressed normally, and that unknown species barriers in mice are still present in the early phase of HIV-1 infection.

HIV-1 preintegration complex (PIC) is composed of newly synthesized viral cDNA and several host and viral proteins, the latter of which include integrase (IN), reverse transcriptase (RT), matrix (MA) and Vpr. The PIC is actively accumulated into the nucleus. We previously showed that the nuclear accumulation of GFP-fused IN (GFP-IN) was significantly reduced in mouse cells than in human cells (Tsurutani et al., 2007), suggesting that inefficient PIC nuclear accumulation in mouse cells might be attributed to the inability of IN to interact with host factors. Lens epithelium-derived growth factor (LEDGF) could be one of such candidates in that it can associate with IN and mediate HIV-1 nuclear accumulation and integration into the chromosome in human cells (Maertens et al., 2003). LEDGF is translated into two proteins, p75 and p52, as a result of alternative splicing (Ge et al., 1998a). LEDGF/p75, but not p52, can associate with IN through its IN-binding domain (IBD; Ge et al., 1998b). Moreover, IN and LEDGF/p75 co-localize in the nucleus of human cells, and recombinant LEDGF/p75 robustly enhances strand transfer activity of IN *in vitro *(Singh et al., 1999). It was also shown that RNAi-mediated knockdown of endogenous LEDGF/p75 abolished IN nuclear accumulation, HIV-1 integration and HIV-1 production (Maertens et al., 2003; Llano et al., 2004b; Ciuffi et al., 2005). LEDGF/p75 is also known as a critical factor for the selection of integration sites such as promoter regions and CpG islands. Thus, LEDGF/p75 has been suggested to be one of important host factors at the PIC nuclear accumulation and integration steps (Bukrinsky, 2004; Llano et al., 2004a, 2006a; Emiliani et al., 2005; Maillot et al., 2013).

In this study, we first created expression plasmids encoding human and mouse LEDGF/p75 (hLEDGF/p75 or mLEDGF/p75, respectively) to compare their ability to support HIV-1 infection in mouse embryonic fibroblast (MEF) cells, and found that the expression of hLEDGF/p75 rendered MEF cells more sensitive to HIV-1 infection than that of mLEDGF/p75. Moreover, we generated hLEDGF/p75 transgenic (hLEDGF/p75 Tg) mice and examined if the expression of hLEDGF/p75 could relieve the species barrier of HIV-1 infection in mouse cells. Transgenic expression of hLEDGF/p75 enhanced IN accumulation in the nucleus and HIV-1 infection in mouse cells, suggesting that LEDGF/p75 is one of the host factors that may determine a species barrier against HIV-1 in mouse cells. We therefore conclude that the transgenic introduction of hLEDGF/p75 would be helpful to generate a small animal model that could be more permissive to HIV-1 infection.

## MATERIALS AND METHODS

### CELLS

HeLa, 293T, and MT4 cells were obtained from ATCC (Rockville, USA), the RIKEN Cell Bank (Ibaraki, Japan), and the Health Science Research Resources Bank (Osaka, Japan), respectively. MEF cells from LEDGF/p75 Knockout mice were kindly provided by A. Engelman (Shun et al., 2007). HeLa, 293T, and MEF cells were maintained in Dulbecco’s modified Eagle’s medium (DMEM; Life Technologies, NY, USA) supplemented with 10% fetal bovine serum (FBS). MT4 cells were maintained in RPMI 1640 medium (Life Technologies, NY, USA) supplemented with 10% FBS.

### PLASMIDS

Env-deficient HIV-1 proviral indicator construct pNL-Luc-E-R+, a vesicular stomatitis virus G (VSV-G)-expressing plasmid pHIT/G, and an HIV-1 NL-Env expression plasmid pNLnΔBS (Tokunaga et al., 1998) were described previously (Fouchier et al., 1997; Tokunaga et al., 2001). The expression vector for a codon-optimized HIV-1 IN fused N-terminally to GFP (GFP-IN) was previously constructed (Tsurutani et al., 2007). The hLEDGF/p75 transgene was constructed as follows. hLEDGF/p75 cDNA was obtained from HeLa cells using SuperScript First-Strand Synthesis System (Invitrogen, CA, USA). A 1.6 kb hLEDGF/p75 fragment was amplified by PCR with KOD-Plus- (TOYOBO, Osaka, Japan). The primer pairs used to amplify hLEDGF/p75 fragments were as follows: forward primer 5′-ACG AAT TCG CCA CCA TGA CTC GCG ATT TCA AAC CTG GAG ACC-3′, reverse primer 5′-CCG AAT TCT CAG TTA TCT AGT GTA GAA TCC TTC AGA GAT ATT TCA G-3′, that have *EcoR*I site, Kozak sequence respectively. PCR product was digested with *EcoR*I and inserted into pCAGGS mammalian expression vector (Niwa et al., 1991). Similarly, C-terminally HA-tagged versions of these plasmids were created by using pCAGGS-3HA expression vector (Iwabu et al., 2009). To generate CD4/CXCR4 expression plasmid, CD4, CXCR4, and ECMV IRES were PCR-amplified from pNL-CD4, pNL-CXCR4 (Tokunaga et al., 2001), and pIRESpuro2 (Clontech, CA, USA), and then digested with *Kpn*I/*Xho*I, *Xho*I/*Not*I, and *Not*I, respectively. Digested fragments were inserted into pCAGGS and the resultant expression plasmid was designated pCa-CD4/CXCR4.

### TRANSFECTIONS AND PROTEIN ANALYSES

5 × 10^5^ 293T cells were transfected with 0.5 μg of either hLEDGF/p75 or mLEDGF/p75 expression plasmid by using FuGENE6 transfection reagent (Promega, Wisconsin, USA), and grown for 48 h. Cell extracts were subjected to gel electrophoresis and then transferred to a nitrocellulose membrane. The membranes were probed with an anti-HA antibody (BD biosciences, NJ, USA). Reacted proteins were visualized by chemiluminescence using an ECL Western blotting detection system (GE Healthcare, Little Chalfont, UK) and monitored using a LAS-3000 imaging system (FujiFilm, Tokyo, Japan).

### INFECTION OF MEF CELLS TRANSIENTLY EXPRESSING LEDGF/p75 WITH HIV-1 REPORTER VIRUSES

2.5 × 10^5^ MEF cells derived from LEDGF/p75 knockout mice were cotransfected with 0.5 μg of either hLEDGF/p75 or mLEDGF/p75 expression plasmid, 0.5 μg of pCa-CD4/CXCR4, and 5 ng of phRL-TK Renilla luciferase expression plasmid (Promega) by using Lipofectamine with Plus reagents (Life Technologies) and grown for 48 h. Infection experiments were performed as described below. Viruses were prepared by cotransfecting 293T cells with 1 μg of pNL-Luc-E-R+, 0.5 μg of pNLnΔBS, and 0.5 μg of an empty plasmid by using FuGENE6. After 48 h, the supernatants were treated with 37.5 units/ml DNase I (Roche Applied Science, MD, USA) for 37°C for 30 min and then harvested, and the amount of p24 antigen was measured by using an HIV-1 p24-antigen capture enzyme-linked immunosorbent assay (ELISA) (Advanced BioScience Laboratories, CA, USA). 1 × 10^4^ MEF cells transiently expressing either hLEDGF/p75 or mLEDGF/p75 were infected with 1 ng of p24 antigen of HIV-1 reporter viruses. At 48 h after infection, cells were lysed with Passive Lysis Buffer (Promega). Cell lysates were subjected to the luciferase assay using the Dual Luciferase Reporter Assay Systems (Promega). Luciferase activity was measured by Centro LB 960 Microplate Luminometer (Berthold Technologies, Bad Wildbad, Germany). Values were normalized by Renilla luciferase activity.

### GENERATION OF hLEDGF/p75 Tg MICE

The hLEDGF/p75 trangene was digested with *BamH*I-*Hind*III, purified from agarose gel with GeneClean Kit (MP-Biomedicals, CA, USA) and adjusted to a final concentration of 5 × 10^5^ copies/μl (2.13 ng/μl). Purified fragments were injected into the male pronuclei of fertilized mouse embryos (C3H/HeN; CLEA Japan Inc, Tokyo, Japan). Mice were kept under specific-pathogen-free conditions in an environmentally controlled clean room at the Center for Experimental Medicine and Systems Biology, the Institute of Medical Science, the University of Tokyo. All experiments were done according to the ethical guidelines for animal experimentation, which was approved by the Institutional Review Board of the Institute of Medical Science, the University of Tokyo.

### SOUTHERN BLOT HYBRIDIZATION

Southern blot analyses were carried out as previously described (Smith and Murphy, 1993). Genomic DNA (10 μg) extracted from the mouse tails was digested with *Pst*I. Digested DNA was electrophoresed and blotted onto a membrane. Membrane was hybridized with ^32^P labeled probe. To detect hLEDGF/p75, an *EcoR*I-*Pst*I (540 bp) fragment from pCAGGS was used as a β-globin probe. The autoradiograms were developed and the band intensity was quantified by a BAS 2000 Bio-Image analyzer (FujiFilm, Tokyo, Japan). Alpha-fetoprotein (AFP) was used as an internal control.

### NORTHERN BLOT HYBRIDIZATION

Total RNA from the thymus, spleen, lymph nodes and thioglycolate (TGC)-elicited macrophages of hLEDGF/p75 Tg mice was prepared by the acid guanidium thiocyanate phenol chloroform method (Chomczynski and Sacchi, 1987). In order to obtain macrophages, mice were injected intraperitoneally with 2 ml of 4% TGC (Difco Laboratories, MI, USA). Three days later, peritoneal exudate cells were collected (Saijo et al., 2007). Northern blot analyses were carried out as previously described (Yasuda et al., 2001). hLEDGF/p75 probe (1.6 kb) was generated by PCR with KOD-Plus- by using the following primers: forward primer; 5′-ACG AAT TCG CCA CCA TGA CTC GCG ATT TCA AAC CTG GAG ACC-3′, reverse primer; 5′-CCG AAT TCT CAG TTA TCT AGT GTA GAA TCC TTC AGA GAT ATT TCA G-3′. The autoradiograms were developed, and the radioactivity of each the bands was quantified with the BAS 2000 Bio-Image analyzer (FujiFilm).

### REAL-TIME RT-PCR

Total RNA from MEF cells was extracted using ReliaPrep RNA Cell Miniprep System (Promega) according to the manufacturer’s instructions. Real-time RT-PCR was performed using One Step SYBR PrimeScript RT-PCR Kit (Takara, Shiga, Japan) with the ABI PRISM 7900HT Sequence Detection System. hLEDGF/p75 mRNA was detected by using the following primers: forward primer; 5′-GAG AAA CAT CAA TGG ATT CTC GAC-3′ and reverse primer; 5′-CTC AAT GCA TCT GTT CAC ATC AAG-3′. GAPDH was detected by using the following primers: forward primer; 5′-GAT GCT GGC GCT GAG TAC G-3′ and reverse primer; 5′-GCA GAG ATG ATG ACC CTT TTG-3′. Levels of hLEDGF/p75 mRNA were normalized with those of GAPDH mRNA.

### PREPARATION OF MEF CELLS EXPRESSING hLEDGF/p75

Mouse embryonic fibroblast cells from hLEDGF/p75 Tg and wild-type (WT) mice were prepared as follows. Fetuses were harvested at 13.5 days of gestation. Embryonic internal organs were removed from the abdominal cavity using dissecting forceps. The embryos were transferred to a 10 cm dish containing 10 ml trypsin/EDTA solution and incubated 20 min at 37°C. After trypsinization, cells were washed and cultured in DMEM. Cell extracts were subjected to gel electrophoresis and then transferred to a nitrocellulose membrane. The membranes were probed with an anti-hLEDGF/p75 antibody (BD biosciences, NJ, USA). Reacted proteins were visualized by chemiluminescence using an ECL Western blotting detection system and monitored using a LAS-3000 imaging system.

### FLUORESCENCE MICROSCOPY

One day prior to transfection, 2 × 10^5^ HeLa, and MEF cells were plated in 8 well culture slides (Nalgene Nunc International, NY, USA) and transfected with 0.8 μg of DNA (GFP-IN) per well using Lipofectamine 2000 (Invitrogen). After 6 h, medium was replaced with fresh culture medium. Forty eight hours post-transfection, cells were washed once in PBS and fixed with acetone for 5 min. After washing with PBS, 1 μg/ml of Hoechst suspended in PBS was added. After 10 min incubation, cells were covered by a coverslip. Confocal microscopy was performed with a Nikon Optiphot-2 fluorescence microscope with a Bio-Rad MRC 1024 laser confocal imaging system (Nikon, Tokyo, Japan).

### INFECTION OF hLEDGF/p75 Tg MEF CELLS WITH VSV-G-PSEUDOTYPED HIV-1 REPORTER VIRUSES

The VSV-G pseudotyped NL4-3-Luc viruses were produced by cotransfecting 293T cells with 1.9 μg of pNL-Luc-E-R+ and 0.1 μg of pHIT/G by using FuGENE6. After 48 h, the supernatants were treated with DNase I for 37°C for 30 min and then harvested, and the amount of p24 antigen was measured by using an HIV-1 p24-antigen capture ELISA. 1 × 10^4^ hLEDGF/p75 Tg MEF cells (No. 089, 110, and 143) were infected with 200 pg of p24 antigen of VSV-G-pseudotyped HIV-1 reporter viruses. At 48 h after infection, cells were lysed with Passive Lysis Buffer and subjected to the luciferase assay. Luciferase activity was measured by Centro LB 960 Microplate Luminometer.

## RESULTS

### FUNCTIONAL DIFFERENCE BETWEEN HUMAN AND MURINE LEDGF/p75 PROTEINS

**FIGURE 1:**
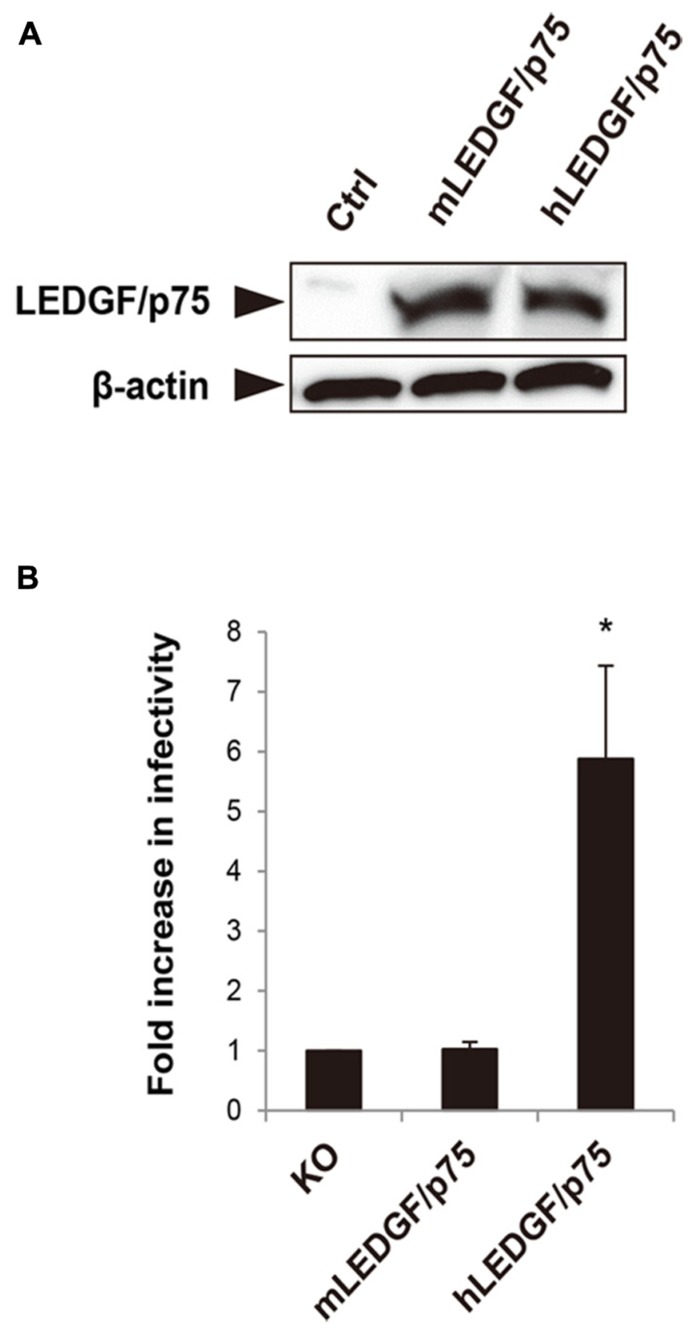
**Comparison of hLEDGF/p75 and mLEDGF/p75 in the ability to support HIV-1 infection. (A)** Detection of hLEDGF/p75 and mLEDGF/p75 proteins. 293T cells were transfected with either hLEDGF/p75 or mLEDGF/p75 expression plasmid and grown for 48 h. Western blot was performed to detect LEDGF/p75 proteins by using an anti-HA antibody. β-actin was used as an internal control. **(B)** HIV-1 infectivity in cells expressing different LEDGF/p75 proteins. MEF cells derived from LEDGF/p75 knockout (KO) mice were cotransfected with either hLEDGF/p75 or mLEDGF/p75 expression plasmid, pCa-CD4/CXCR4, and phRL-TK Renilla luciferase expression plasmids, and grown for 48 h. MEF cells transiently expressing either hLEDGF/p75 or mLEDGF/p75 were infected with HIV-1 reporter viruses. At 48 h after infection, cells were subjected to the luciferase assay. Values were normalized with Renilla luciferase activity. *P < 0.01 (*t*-test).

We previously showed that the nuclear accumulation of PIC was blocked in mouse cells (Tsurutani et al., 2007), suggesting that mLEDGF/p75 might be defective at this step. To test this hypothesis, we created HA-tagged hLEDGF/p75 and mLEDGF/p75 expression plasmids. Protein expressions in the cells transfected with each plasmid were confirmed by immunoblotting using anti-HA antibodies (**Figure 1A**). We cotransfected MEF cells derived from LEDGF/p75 knockout mice with either hLEDGF/p75 or mLEDGF/p75, together with CD4/CXCR4 expression plasmid. We then examined the efficiency of HIV-1 infection by using MEF cells transiently expressing hLEDGF/p75 or mLEDGF/p75. We found that infection was more efficient in MEF cells expressing hLEDGF/p75 than those expressing mLEDGF/p75 (**Figure 1B**). These results suggest that hLEDGF/p75 expression renders MEF cells more susceptible to HIV-1 infection.

### GENERATION OF TRANSGENIC MICE CARRYING THE hLEDGF/p75

**FIGURE 2:**
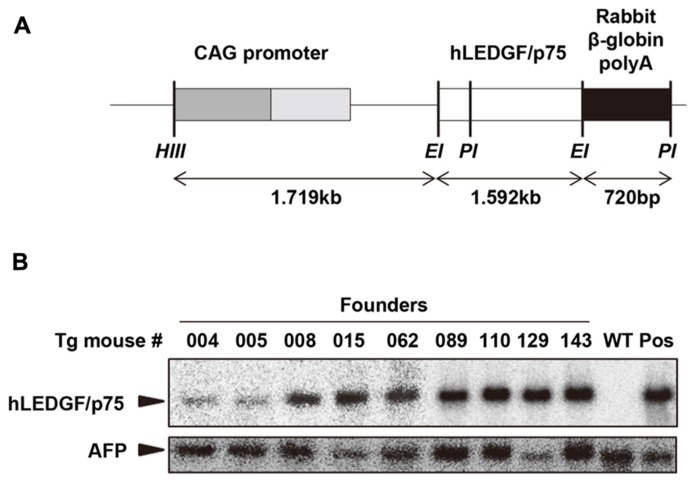
**Generation of hLEDGF/p75 Tg mice. (A)** Construction of the transgene. hLEDGF/p75 gene (white box) was ligated downstream of the CMV-immediate-early enhancer/chicken β-actin promoter (CAG) promoter. Restriction enzyme sites are indicated as follows: *Hind*III (HIII), *EcoR*I (EI) and *Pst*I (PI). **(B)** Detection of the transgene in hLEDGF/p75 Tg mice by Southern blot hybridization analysis. Cellular DNA (10 μg) was extracted from the mouse tails, digested with *PstI*, and was electrophoresed in an agarose gel. β-globin poly A was used as a probe (black box). Cellular DNA from WT mouse was used as a negative control (WT), and WT DNA containing pCAGGS-hLEDGF/p75 plasmid copies was used as a positive control (Pos). Alpha-fetoprotein (AFP) was used as an internal control. The sizes expected for hLEDGF/p75 and AFP are indicated by arrowheads.

We generated Tg mice carrying the hLEDGF/p75 to examine whether the host factor would be able to confer HIV-1 susceptibility to the animals. To obtain Tg mice expressing high levels of hLEDGF/p75 in multiple tissues, a transgene consisting of the cytomegalovirus immediate-early enhancer/chicken β-actin promoter, the hLEDGF/p75 cDNA and rabbit β-globin poly A, was constructed (**Figure 2A**). Transgenic founders were obtained by microinjecting the transgene into the pronuclei of fertilized embryos from WT parents (C3H/HeN). Southern blot hybridization was carried out to detect the transgene by using a probe specific for β-globin polyA. Out of 165 offspring, we obtained 9 transgenic founder mice in which the hLEDGF/p75 transgene was integrated (**Figure 2B**).

### hLEDGF/p75 TRANSGENE EXPRESSION IN hLEDGF/p75 Tg MICE

**FIGURE 3:**
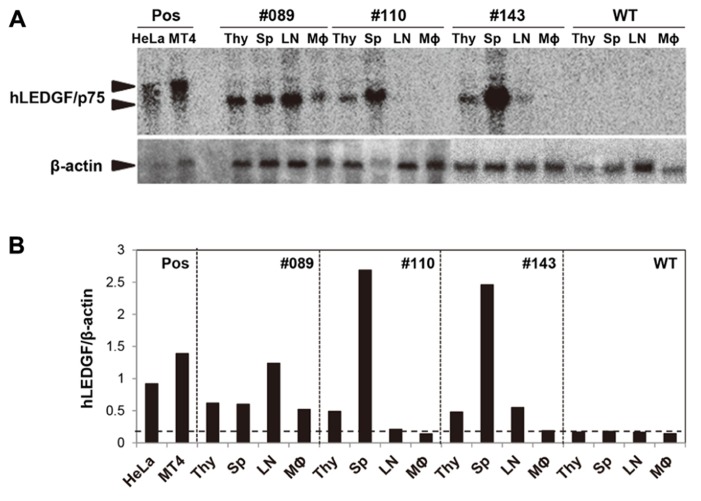
**hLEDGF/p75 expression in Tg mice. (A)** Total RNA was extracted from the thymus (Thy), spleen (Sp), lymph-nodes (LN), and TGC-elicited macrophages from hLEDGF/p75 Tg mice. Northern blot hybridization was carried out using 10 μg of RNA prepared from tissues. hLEDGF/p75 cDNA was used as a probe, and β-actin was used as an internal control. RNA from the WT mouse and from HeLa and MT4 cells were used as a negative control (WT), and as positive controls (Pos), respectively. The sizes expected for hLEDGF/p75 and β-actin are indicated by arrowheads. **(B)** The band intensity in **(A)** was measured with a BAS 2000 Bio-Image analyzer. Expression levels of hLEDGF/p75 relative to those of β-actin are shown for each transgenic line. The threshold is indicated by a horizontal dotted line.

In order to analyze the expression of hLEDGF/p75 mRNA in hLEDGF/p75 Tg F1 mice, Northern blot hybridization was performed by using total RNAs purified from thymus, spleen, lymph-nodes, and TGC-elicited macrophages. As shown in **Figure 3A**, 1.7 kb bands were detected in hLEDGF/p75 Tg mice, while the endogenous 2.1 kb bands were detected in HeLa and MT4 cells. hLEDGF/p75 mRNA from line No. 089 Tg mouse was detected in all the tissues, in which levels of hLEDGF/p75 expression was similar to those in HeLa or MT4 cells. The mRNA was also present in the thymus and spleen derived from the line No. 110 Tg mouse, in the thymus, spleen and lymph-nodes of the line No. 143 Tg mouse ( **Figures 3A,B**). Either the line No. 004, 005, 008, 015, and 062 Tg mice or the line No. 129 Tg mice expressed only in the thymus or lymph-nodes, respectively (data not shown). For further studies, we thus selected 3 lines (line No. 089, 110, and 143 Tg mice) expressing relatively high hLEDGF/p75 mRNA in the thymus, spleen and lymph nodes.

### ESTABLISHMENT OF MEF CELLS DERIVED FROM hLEDGF/p75 Tg MICE

**FIGURE 4:**
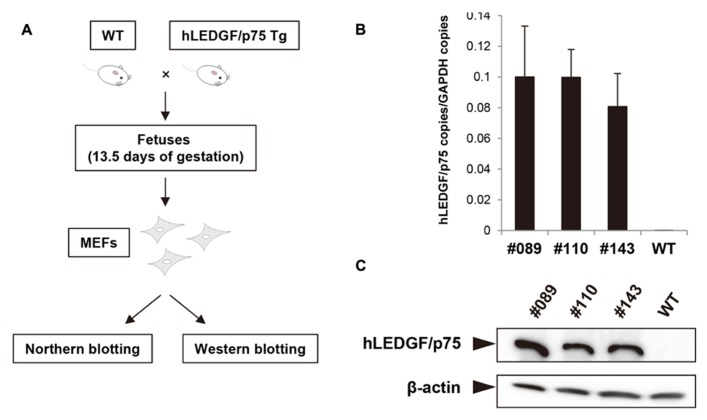
**Preparation of MEF cells and hLEDGF/p75 expression in MEF cells. (A)** MEF cells from hLEDGF/p75 Tg and WT mice were prepared from mice fetuses. Mice were mated with WT mice, and the resulting fetuses were harvested at 13.5 days of gestation. Embryonic internal organs were removed from the abdominal cavity. The embryos were dissociated and trypsinized to produce single-cell suspensions. After expansion, the expression of hLEDGF/p75 in MEF cells was analyzed. **(B)** Real-time RT-PCR was performed to evaluate the hLEDGF/p75 mRNA expression in hLEDGF/p75 Tg MEF cells by using hLEDGF/p75 specific primers. The copy numbers were normalized with that of GAPDH. **(C)** Western blotting was carried out using proteins from Tg and WT MEF cells. An anti-hLEDGF/p75 antibody was used to detect the protein, and β-actin was detected as an internal control.

To investigate whether hLEDGF/p75 promotes IN nuclear accumulation, we examined the subcellular localization of IN in hLEDGF/p75 Tg mouse cells. We generated MEF cells from hLEDGF/p75 Tg mice and the expression of hLEDGF/p75 was examined. As shown in **Figure 4A**, MEF cells from hLEDGF/p75 Tg mice were prepared from mice fetuses. Tg mice were mated with WT, and fetuses were harvested at 13.5 days of gestation and removed embryonic internal organs from the abdominal cavity. The embryos were dissociated and trypsinized to produce single-cell suspensions. After expansion, we confirmed that hLEDGF/p75 mRNA (**Figure 4B**) as well as hLEDGF/p75 protein (**Figure 4C**) was detected in MEF cells from the line No. 089, 110, and 143 Tg mice.

### ENHANCED NUCLEAR ACCUMULATION OF GFP-IN IN MEF CELLS FROM hLEDGF/p75 Tg MICE

**FIGURE 5:**
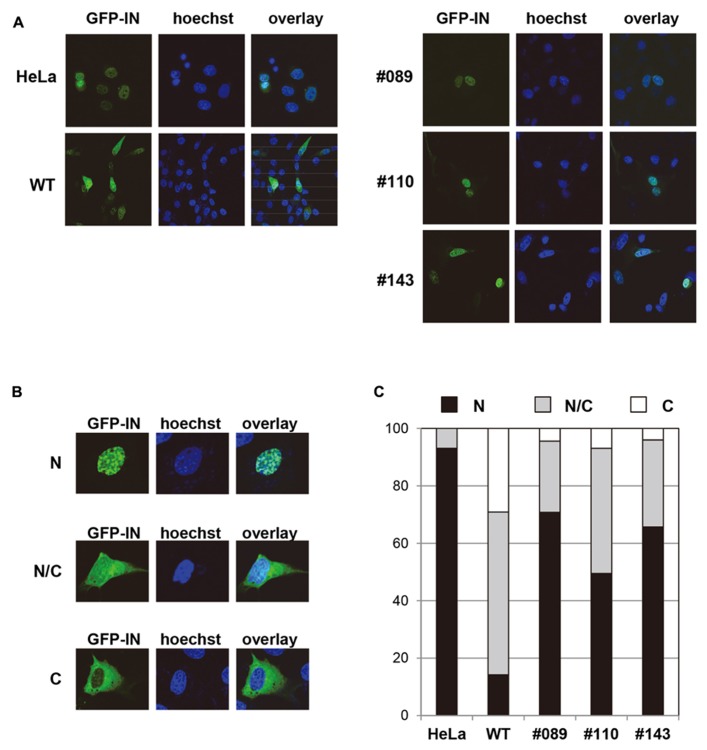
**Subcellular localization of GFP-IN in MEF cells. (A)** HeLa and MEF cells were transfected with a plasmid expressing GFP-IN (green). After 48 h, cells were fixed and visualized by a confocal fluorescence microscopy. A typical distribution of GFP fluorescence is shown in MEF cells from different Tg lines. Hoechst (blue) was used to stain nuclear DNA. HeLa and WT MEF cells were used as a positive and negative control, respectively. **(B)** GFP positive cells were classified as follows; N, the fluorescence of GFP-IN is higher in the nucleus than in the cytoplasm; N/C, the fluorescence of GFP-IN in the nucleus is similar to the cytoplasm; and C, the fluorescence of GFP-IN is higher in the cytoplasm than in the nucleus. **(C)** GFP positive cells classified by (B) were quantified by counting approximately 100 cells.

HeLa cells, and MEF cells derived from either WT or hLEDGF/p75 Tg mice (line No. 089, 110, and 143) were transfected with a plasmid expressing codon-optimized IN that was N-terminally fused to GFP, and the subcellular localization was examined by fluorescence and confocal microscopy. As shown in **Figure 5A**, we observed the accumulation of GFP-IN in the nucleus of hLEDGF/p75 Tg-derived MEF cells, making a clear contrast to those from WT mice. To quantitatively evaluate the efficiency of nuclear accumulation of GFP-IN, we classified the cells as follows; (N) the fluorescence of GFP-IN is higher in the nucleus than in the cytoplasm; (N/C) the fluorescence of GFP-IN in the nucleus is similar to the cytoplasm; and (C) the fluorescence of GFP-IN is higher in the cytoplasm than in the nucleus (**Figure 5B**). As expected, GFP-IN preferentially localized mainly into the nucleus in HeLa cells (N: 93.1%), while GFP-IN did not localize exclusively in the nucleus in WT MEF cells (N: 14.2%) but mainly in both the cytoplasm and the nucleus in WT MEF cells (N/C: 56.7%). In contrast, GFP-IN significantly accumulated in the nucleus in MEF cells from line No. 089, 110, and 143 Tg mice (N: 70.8, 49.5, and 65.7, respectively; **Figure 5C**). These data suggest that hLEDGF/p75 enhances IN accumulation into the nucleus of mouse cells, probably by stably tethering the viral protein to chromatin as previously reported (Maertens et al., 2003; Llano et al., 2004b; Emiliani et al., 2005; Vanegas et al., 2005; Turlure et al., 2006).

### THE SUSCEPTIBILITY OF hLEDGF/p75 Tg MEF CELLS TO HIV-1 INFECTION

**FIGURE 6:**
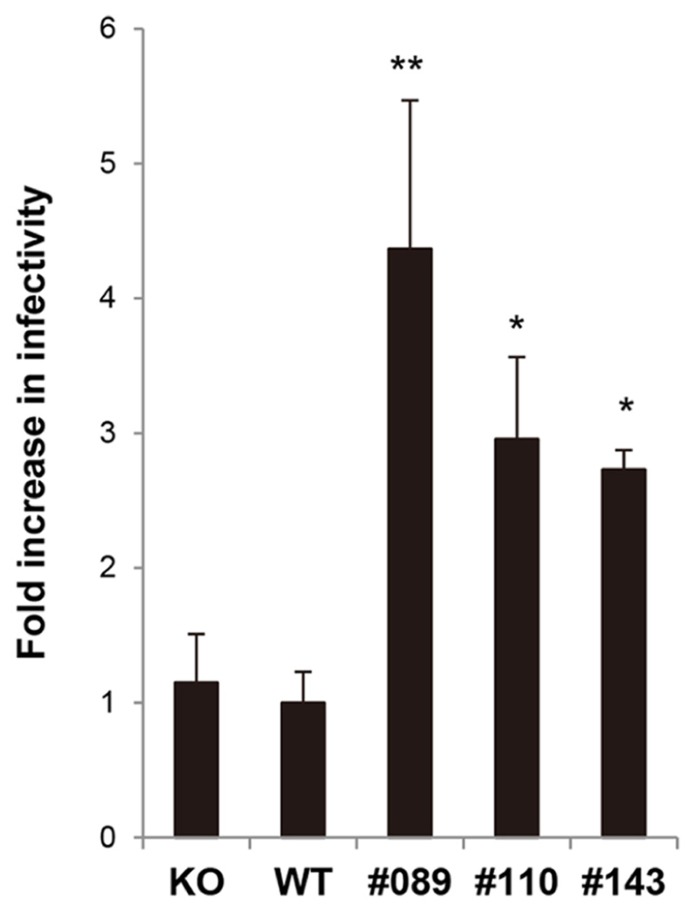
**Susceptibility of hLEDGF/p75 Tg MEF cells to HIV-1 infection.** MEF cells derived from hLEDGF/p75 Tg, WT and LEDGF knockout (KO) mice were infected with an equivalent dose of VSV-G pseudotyped HIV-1 luciferase reporter virus. After 48 h, cells were lysed and subjected to luciferase assay. The data shown are the mean ± SD of triplicate experiments.*P < 0.05, **P < 0.01 (*t-*test).

Finally, we examined the susceptibility of MEF cells from hLEDGF/p75 Tg mice to HIV-1 infection. Tg (line No. 089, 110, and 143), WT, and LEDGF/p75 knockout MEF cells were infected with VSV-G-pseudotyped NL4-3 viruses, and then compared the efficiency of HIV-1 infection. We found that hLEDGF/p75 Tg MEF cells were significantly more susceptible to HIV-1 than WT and mLEDGF/p75 knockout MEF cells (**Figure 6**). It should be noted that the poor susceptibility of WT MEF cells to HIV-1 infection was almost equivalent to that of the mLEDGF/p75 knockout cells (**Figure 6**, left bar). Taken altogether, we conclude that hLEDGF/p75 is able to relieve the species barrier against HIV-1 infection in mouse cells by supporting HIV-1 PIC accumulation into the nucleus.

## DISCUSSION

Pleiotropic functions of hLEDGF/p75 were suggested in HIV-1 infection including promotion of nuclear accumulation of PIC (Cherepanov et al., 2003, 2004; Maertens et al., 2003; Llano et al., 2004b; Ciuffi et al., 2005; Vanegas et al., 2005; Van Maele et al., 2006), selection of HIV-1 integration sites and enhancement of proviral integration (Bukrinsky, 2004; Llano et al., 2004a, 2006a; Emiliani et al., 2005). We previously showed that one of the species barriers resides at the PIC nuclear accumulation step in mouse cells and that this restriction was caused by a dysfunction of the IN-dependent PIC accumulation system (Tsurutani et al., 2007).

In this study, we have generated 9 Tg mouse lines carrying hLEDGF/p75, to elucidate the role of LEDGF/p75 in the nuclear accumulation of IN. Among them, mice from line No. 089, 110, and 143 expressed hLEDGF/p75 in the thymus, spleen, lymph nodes and MEF cells. We found that the nuclear accumulation of HIV-1 IN in hLEDGF/p75 Tg MEF cells was greatly enhanced. Under these assay conditions, GFP-IN was less frequently localized to the nucleus in WT MEF cells. These results are consistent with the efficiency of luciferase expression after infection with HIV-1 pseudoviruses carrying a luciferase gene (**Figure 6**), suggesting that LEDGF/p75 is one of the host factors responsible for the HIV-1 species barrier.

The homology between hLEDGF/p75 and mLEDGF/p75 is high (92.3% amino acid identity). LEDGF/p75 is composed of four functional domains, the N-terminal Pro-Trp-Trp-Pro domain (residues 1–93), the C-terminal IN-binding domain (residues 347–429), the nuclear localization signal and the AT-hook DNA-binding motif (Maertens et al., 2003; Cherepanov et al., 2004, 2005; Vanegas et al., 2005; Llano et al., 2006a,b). The amino acid identity of these domains and motifs are completely matched between mouse and human LEDGF/p75. Therefore, it is likely that the species barrier in mouse cells might be caused by only small structural differences of previously underappreciated regions between mouse and human LEDGF/p75 proteins.

Shun et al. (2007) previously reported that HIV-1 integration was severely reduced in mLEDGF/p75 knockout mouse cells, suggesting that mLEDGF/p75 is able to support HIV-1 infection. In contrast, our data showed that the level of viral infectivity in WT MEF cells was almost equivalent to that in mLEDGF/p75 knockout cells used in the above report (Shun et al., 2007), rather suggesting that the mouse version might not contribute to the infection (**Figure 6**). This discrepancy might be due to the fact that our WT MEF cells are derived from the C3H/HeN mouse strain while mLEDGF/p75 knockout cells are derivatives of C57BL/6 mice. This needs to be elucidated with further experiments by comparison of different mouse strains. It should be noted that strain-specific epigenetic differences in mice, such as methylation patterns, have recently been reported (Schilling et al., 2009).

Small animal models for HIV-1 infection such as *Rag2^-/-^/Il2rg^-/-^* mice (Traggiai et al., 2004) and BLT mice (Denton et al., 2008) have made significant contributions to our understanding of HIV/AIDS pathogenesis. However, the former mice show insufficient induction of an immune response against HIV-1 (Baenziger et al., 2006; An et al., 2007), while the use of latter mice has been influenced by some ethical restrictions and limited availability. On the other hand, immunologically intact transgenic mouse models are relatively straightforward and inexpensive, in which high levels of target gene expression can be easily achieved resulting in an obvious phenotype. 

To generate humanized mouse models for HIV-1 infection, so far we have generated hCD4/hCXCR4/hCycT1 Tg mice and hCD4/hCCR5/hCycT1 Tg mice (Tsurutani et al., 2007). The addition of hLEDGF/p75 to these Tg mice should increase the susceptibility of these mice to HIV-1 infection, especially during the early phase of infection. However, we also need to pay attention to other host factors that restrict HIV-1 infection in mice, such as APOBEC3, which is an APOBEC-related cytidine deaminases (Kobayashi et al., 2004), because these inhibitors are also active in mouse cells (Yu et al., 2003; Kobayashi et al., 2004; Mous et al., 2012). Further characterization and identification of factors involved in host range barriers that are also present in the late phase of the viral replication cycle (transcription, RNA, export, and virion budding) should provide a new insight into the molecular mechanisms of HIV-1 replication and clues to the development of new therapeutics.

## Conflict of Interest Statement

The authors declare that the research was conducted in the absence of any commercial or financial relationships that could be construed as a potential conflict of interest.

## AUTHOR CONTRIBUTIONS

Takuya Tada performed the experiments, analyzed the data and wrote the paper. Motohiko Kadoki analyzed the data. Yang Liu performed the experiments. Kenzo Tokunaga supervised the research, analyzed the data, and wrote the paper. Yoichiro Iwakura designed the study, supervised the work, analyzed data, and wrote the paper.
